# Supplementary material for: Tax preference, financing constraints and enterprise investment efficiency—Experience, of China’s enterprises investment
Source: PLoS One. 2022 Sep 22;17(9):e0274336. doi: 10.1371/journal.pone.0274336 (PMC9499234; doi:10.1371/journal.pone.0274336)
Supplement: Attachment — Submitted filename: Response to Reviewers.doc [file pone.0274336.s010.doc]

**Original Article Title:** “Tax Preference, Financing Constraints and Enterprise Investment Efficiency——Experience of China’s Enterprises Investment”

**To:** Editor Ali Safaa Sadiq

**Re:** Response to Editor

Dear Editor,

Thank you for allowing a resubmission of our manuscript, with an opportunity to address the comments.

Best regards,

Yujing Feng

**Response to editor:**

**Editor, Journal Requirements# 1:** Please review your reference list to ensure that it is complete and correct. If you have cited papers that have been retracted, please include the rationale for doing so in the manuscript text, or remove these references and replace them with relevant current references. Any changes to the reference list should be mentioned in the rebuttal letter that accompanies your revised manuscript. If you need to cite a retracted article, indicate the article’s retracted status in the Reference.

**Author response:** We checked each paper in the reference list individually and found that the information in two papers was inaccurate.

1. The third reference is a working paper. In the original manuscript, the year was mistakenly written as 1988, which has now been changed to the correct year of 1987.

2. The year is omitted in the 29th reference, which has been supplemented.

Other than that, no retracted references were found. If editors or experts find such a situation, please let us know in detail.

**Author, Request for Modification of Funding Information:**

**Author:**

Our Funding Information has been changed to remove "The major project of the National Social Science Foundation of China "Research on China's Solutions for Anti-Globalization Trends and the Reconstruction of International Economic and Trade Rules" (17ZDA097)", and added "Youth Project of Natural Science Foundation of Anhui Province, China. "Economic Policy Uncertainty and Servicization of Manufacturing Enterprises: Theoretical Framework, Impact and Identification of Causal Effects" (2208085QG222)". So the new Funding Information list is as follows:

Funding Information:

The key project of the National Social Science Foundation of China, "Analysis of the Political Economy of 'Reverse Globalization' and Research on China's Countermeasures" (18AGJ001).

Anhui Province University Scientific Research Key Project (Humanities and Social Sciences) "Reform of Security Property Rights System, Financing Constraints and Domestic Value-Added Rate of Exports of Manufacturing Enterprises" (SK2021A0224)

Youth Project of Natural Science Foundation of Anhui Province, China. "Economic Policy Uncertainty and Servicization of Manufacturing Enterprises: Theoretical Framework, Impact and Identification of Causal Effects" (2208085QG222)

**Response to reviewer 2:**

**Reviewer # 2, Concern # 1:** The results of your comparative study should be discussed in-depth and with more insightful comments on the behaviour of your algorithm on various case studies. Discussing results should not mean reading out the tables and figures once again.

**Author response:** We have conducted a more in-depth analysis of the empirical results in the second paragraph of the Further expand the analysis section of the original manuscript, and for further depth, we have added the following.

“This policy aims to reduce the income tax burden in the early stage of investment, promote enterprises to speed up the renewal of machinery and equipment, and stimulate research and development innovation. The emergence of over-investment may be due to the low efficiency of resource allocation within the enterprise, or even the existence of resource misallocation, and the lag in the upgrading of human capital structure, which cannot match suitable human capital for newly increased fixed asset investment. Therefore, the investment made by the enterprise under the incentive of the policy can easily become the repeated investment, the production potential cannot be fully tapped, the equipment upgrade cannot produce the technology spillover effect, and the productivity and R&D innovation of the enterprise cannot be improved accordingly. ”

**Reviewer # 2, Concern # 2:** Avoid lumping references as in [x, y] and all other. Instead summarize the main contribution of each referenced paper in a separate sentence. For scientific and research papers, it is not necessary to give several references that say exactly the same. Anyway, that would be strange, since then what is innovative scientific contribution of referenced papers? For each thesis state only one reference.

**Author response:** In order to understand our research on this topic in as much detail as possible, we have read a large number of references, and it is very common that many literatures have been studied from different angles or methods and have reached the same point of view or conclusion. So it is unavoidable to have multiple reference marks after the same idea. We also checked recent papers published in the journal of PLOS ONE, many of which have a similar situation. Therefore we have not made changes here.

**Reviewer # 2, Concern # 3:** Avoid using first person.

**Author response:** We accept comments, and have revised all first-person descriptions.

**Reviewer # 2, Concern # 4:** Avoid using abbreviations and acronyms in title, abstract, headings and highlights.

**Author response:** We have supplemented the abbreviations in the abstract with the full name. We have paid attention to this in the process of writing the paper, and the full name of abbreviations will be displayed for the first time in the paper. However, if you write the full name directly in the title of the chapter, it will cause the title to be too long and affect the appearance. But we are sure that before the title appears abbreviations, the full name of the abbreviations has already been explained in the previous text.

**Reviewer # 2, Concern # 5:** Please avoid having heading after heading with nothing in between, either merge your headings or provide a small paragraph in between.

**Author response:** In order to avoid this situation, we moved Table 2 to the bottom of the analysis content to avoid the connection between the header and the title. The titles of other individual chapters and section titles are next to each other, referring to the structure of articles published in the journal of PLOS ONE.

**Reviewer # 2, Concern # 6:** The first time you use an acronym in the text, please write the full name and the acronym in parenthesis. Do not use acronyms in the title, abstract, chapter headings and highlights.

**Author response:** We accept comments and have made adjustments in the original paper. We have supplemented the full name and the acronym in parenthesis. For example, the full names of PSM-DID and RD are supplemented in the abstract. Added the full name of ST that appears in the last paragraph of the section Sample selection and data sources.

research and development (R&D)

Propensity Score Matching – Difference in Differences (PSM-DID)

ST (Special Treatment)

**Reviewer # 2, Concern # 7:** The results should be further elaborated to show how they could be used for the real applications.

**Author response:** We added further details at the end of the Conclusion section to show how they can be used in practical applications.

**“**In general, the current problem faced by Chinese investment is that the scale is large but the efficiency is too low, which also severely limits the high-quality development of the Chinese economy. Investment has always been one of the troikas driving China's economic growth, so the government has continued to introduce various policies to stimulate it. The results of this paper show that policies that stimulate investment do not necessarily improve investment efficiency at the same time, or even inhibit the improvement of investment efficiency. The accelerated depreciation policy of event fixed assets selected in this paper has a very clear boundary between supported industries and unsupported industries, and it is easy to distinguish affected industries from unaffected industries. Moreover, the buffer period from policy introduction to implementation is short, which can be regarded as an exogenous event for individual enterprises. These all create good preconditions for the application of the difference in differences model. The implementation of this policy has caused efficiency losses to corporate investment, mainly because companies are prone to over-investment under the stimulus of the policy. Enterprises should accelerate R&D investment and technological innovation, and improve the efficiency of resource allocation, in order to give full play to the policy advantages, and enterprises can also obtain actual benefits.”

All modifications were made using the MS Word Track Changes function.
