# Supplementary material for: Tax preference, financing constraints and enterprise investment efficiency—Experience, of China’s enterprises investment
Source: PLoS One. 2022 Sep 22;17(9):e0274336. doi: 10.1371/journal.pone.0274336 (PMC9499234; doi:10.1371/journal.pone.0274336)
Supplement: Attachment — Submitted filename: Response to Reviewers.doc [file pone.0274336.s011.doc]

**Original Article Title:** “Tax Preference, Financing Constraints and Enterprise Investment Efficiency——Experience of China’s Enterprises Investment”

**To:** Editor Ali Safaa Sadiq

**Re:** Response to Editor

Dear Editor,

Thank you for allowing a resubmission of our manuscript, with an opportunity to address the comments.

Best regards,

Yujing Feng

**Response to editor:**

**Request for Modification of Funding Information**

**Author:**

We confirm that the *Funding Information list* is as follows:

Funding Information:

The key project of the National Social Science Foundation of China, "Analysis of the Political Economy of 'Reverse Globalization' and Research on China's Countermeasures" (18AGJ001).

Anhui Province University Scientific Research Key Project (Humanities and Social Sciences) "Reform of Security Property Rights System, Financing Constraints and Domestic Value-Added Rate of Exports of Manufacturing Enterprises" (SK2021A0224)

Youth Project of Natural Science Foundation of Anhui Province, China. "Economic Policy Uncertainty and Servicization of Manufacturing Enterprises: Theoretical Framework, Impact and Identification of Causal Effects" (2208085QG222)

**Response to reviewer 2:**

**Reviewer # 2, Concern # 1:** We confirm that all the images used in this work copyrights are free.

**Author response:** We have

**Reviewer # 2, Concern # 2:** Also, the list of references should be carefully checked to ensure consistency with between all references and their compliances with the journal policy on referencing.

**Author response:** We have revised the format of the references to the style required by the journal.

All modifications were made using the MS Word Track Changes function.
